# Evaluation of Thawing and Stress Restoration Method for Artificial Frozen Sandy Soils Using Sensors

**DOI:** 10.3390/s21051916

**Published:** 2021-03-09

**Authors:** Jongchan Kim, Jong-Sub Lee, Cody Arnold, Sang Yeob Kim

**Affiliations:** 1 Department of Civil and Environmental Engineering, University of California at Berkeley, Berkeley, CA 94720, USA; jkim3139@berkeley.edu; 2 School of Civil, Environmental and Architectural Engineering, Korea University, Seoul 02841, Korea; jongsub@korea.ac.kr; 3 School of Civil and Environmental Engineering, Georgia Institute of Technology, Atlanta, GA 30332, USA; cody.arnold@gatech.edu

**Keywords:** artificial ground freezing, frozen soils, liquefaction, stress restoration, thawing

## Abstract

Undisturbed frozen samples can be efficiently obtained using the artificial ground freezing method. Thereafter, the restoration of in situ conditions, such as stress and density after thawing, is critical for laboratory testing. This study aims to experimentally explore the effects of thawing and the in situ stress restoration process on the geomechanical properties of sandy soils. Specimens were prepared at a relative density of 60% and frozen at −20 °C under the vertical stress of 100 kPa. After freezing, the specimens placed in the triaxial cell underwent thawing and consolidation phases with various drainage and confining stress conditions, followed by the shear phase. The elastic wave signals and axial deformation were measured during the entire protocol; the shear strength was evaluated from the triaxial compression test. Monotonic and cyclic simple shear tests were conducted to determine the packing density effect on liquefaction resistance. The results show that axial deformation, stiffness, and strength are minimized for a specimen undergoing drained thawing, restoring the initial stress during the consolidation phase, and that denser specimens are less susceptible to liquefaction. Results highlight that the thawing and stress restoration process should be considered to prevent the overestimation of stiffness, strength, and liquefaction resistance of sandy soils.

## 1. Introduction

With the recent increase in demand for the complicated and extensive infrastructure, construction is being conducted on comparatively weak deposits, such as loosely compacted granular sand. The undrained shear response of the deposits, which is one of the most important geomechanical characteristics, is primarily affected by the soil fabric, density, and stress conditions [1]. However, mimicking the in situ fabric, including aging and cementation, is challenging in the laboratory [2]. The most effective method for evaluating engineering parameters (i.e., the in situ strength and deformation characteristics under static and dynamic loading conditions) can be achieved through laboratory tests using undisturbed specimens. Thus, considerable attention has been focused on obtaining undisturbed samples from sand deposits to evaluate soil characteristics (e.g., log information, shear strength, and liquefaction resistance).

Generally, an undisturbed sandy sample cannot be obtained using conventional sampling methods. In particular, the lack of cohesion and capillary pressure between particles may cause numerous difficulties in maintaining an undisturbed condition during coring. Recently, the artificial ground freezing method is considered to be the most effective technique for obtaining undisturbed soil samples from cohesionless sandy deposits [3,4,5,6]. However, the ground freezing process should avoid volume expansion associated with a 9% increase in pore-water volume during freezing. Therefore, the freezing rate should be controlled to expel the excess volume of water from the opposite side of the freezing front.

The frozen specimen obtained using the artificial ground freezing system should be thawed and recovered to the initial stress conditions for further laboratory testing. Any disturbance during the handling of the frozen specimens and testing should be managed to minimize disruption of the specimen [2]. Several studies have been conducted regarding the thawing and stress restoration methods of undisturbed frozen specimens. Drained triaxial compression tests have been conducted using undisturbed frozen specimens and reconstituted unfrozen specimens, in which the freeze-thaw process was determined not to significantly affect the strength and deformation of sandy soils [7]. Hofmann et al. [4] conducted an alternative thawing method with frozen specimens obtained from a depth of 28–37 m, and attempted to determine the applicable thawing method according to the direction of thawing and the stress restoration point of the in situ stress conditions. The author suggested that applying 90% of the in situ stress during unidirectional thawing causes fewer changes in the void ratio than during multidirectional thawing. However, unidirectional thawing cannot be entirely maintained in the laboratory owing to the inevitable radial direction thawing [2]. Researchers proposed that multidirectional thawing causes greater deformation under compressive conditions owing to the frozen kernel behaving as a stress concentrator [8]; in cases where higher effective stress is applied to the multidirectional thawing specimens, a greater sample disturbance is expected. Therefore, more practical suggestions and evaluations of the thawing and stress restoration methods of frozen specimens for laboratory tests are required.

This study experimentally explores the methods of thawing and the in situ stress recovery protocols of frozen sandy soils. The frozen specimens were prepared in the laboratory using a customized freezing mold, and different drainage conditions during the thawing and stress restoration methods were applied. During the triaxial compression test, the P- and S-wave velocities and axial deformation were measured to evaluate the various test protocols in terms of the sample disturbance. In addition, a series of monotonic/cyclic simple shear tests were conducted to estimate the effects of the packing density of sandy soil, which may be affected by the thawing and stress restoration methods for artificial frozen soils, on the liquefaction resistance. The results provide an optimal test protocol to minimize sample disturbance during the thawing and restoration process of in situ stress.

## 2. Materials and Methods

### 2.1. Specimens Preparation

**Figure 1:**
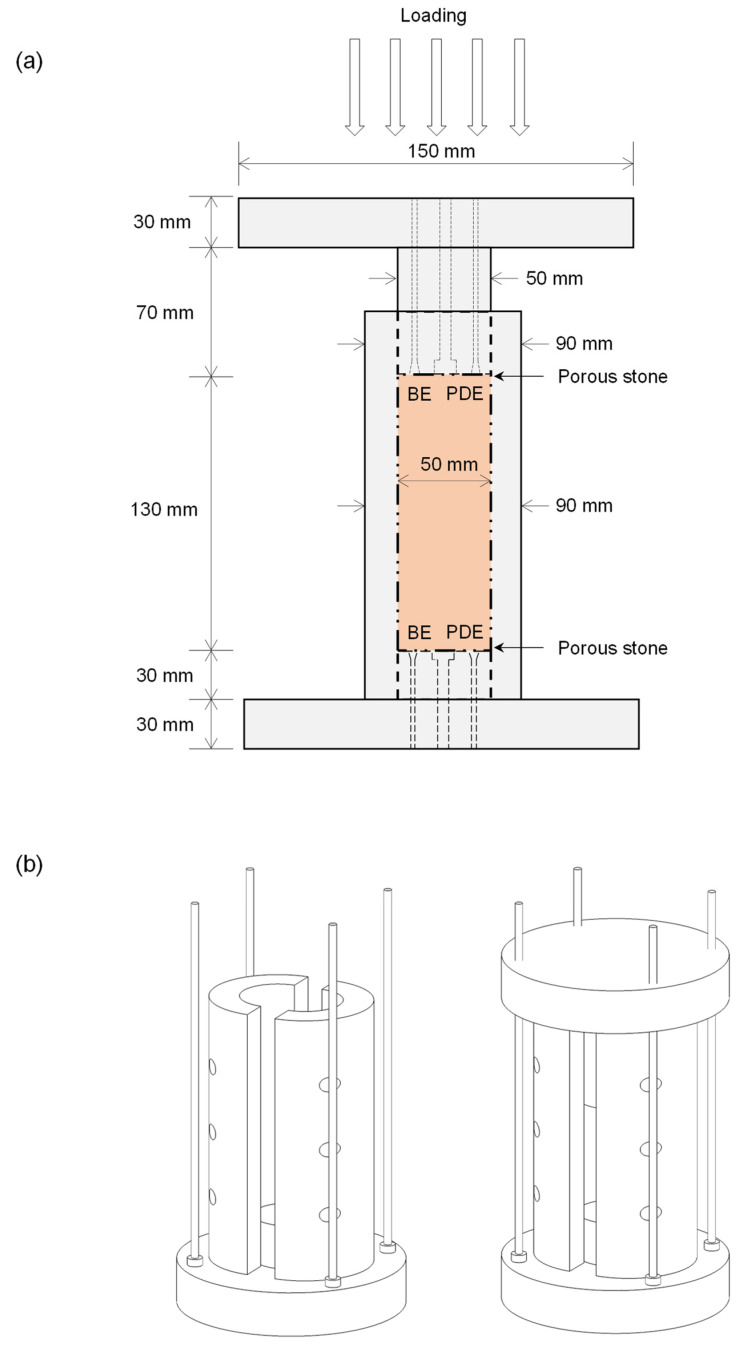
Schematic drawings of the freezing mold. Insulation materials are covered, except the bottom of the mold, to ensure unidirectional freezing (i.e., bottom to top). The volume change of pore water caused by freezing can be expelled through the top and bottom drainage. (**a**) Cross-sectional drawing with dimensions. (**b**) Three-dimensional drawing before and after assembling.

Figure 1 illustrates a schematic drawing of the freezing mold designed to produce the frozen specimens in the laboratory. The specimens were frozen in the laboratory to control the surcharging loading and drainage conditions during freezing. The freezing mold was made of aluminum (AL6061). It consists of top/bottom caps and a cylindrical main body that is longitudinally disassembled into two parts (Figure 1b). Two porous stones were placed on the top and bottom of the specimens during freezing to allow uniform pore-water drainage. A pair of piezo disk elements (PDEs) and bender elements (BEs) are equipped on the top and bottom caps to capture the P- and S-wave signals. The specimen can be subjected to the target vertical stress by surcharging the loading on the top plate. The top and bottom plates were connected using four guide screws for a straight alignment between the top and bottom caps. The top plate can move vertically to respond to the volume change of the specimen. The freezing mold was submerged in a water bath to ensure that the specimens were fully saturated.

The specimens were prepared using Jumunjin Sand (Jumunjin Silica Sand Co., Jumunjin, Korea), passing the No. 30 sieve (0.60 mm) and being retained in the No. 50 sieve (0.30 mm), to minimize the effects of size and the swelling of the specimens during freezing. The mean particle size *D_50_* was 0.45 mm, and the maximum and minimum void ratios were *e_max_*/*e_min_* = 0.96/0.67 [9,10,11]. The specific gravity, *G_s_*, of the sand was 2.66.

The sand was packed into a customized freezing mold under dry conditions with a relative density of 60%, after which de-aired water was added to the water bath for upward water flow through the specimens. A weight corresponding to a 100 kPa vertical stress was applied to the specimen. The insulation materials covered the upper part of the freezing mold to induce upward freezing. When unidirectional freezing is attained, the volume expansion caused by the pore-water phase change can be expelled through the drainage lines. The freezing mold and water bath were placed in a walk-in freezing chamber, and the temperature was lowered and maintained at −20 °C for 60 h.

**Figure 2:**
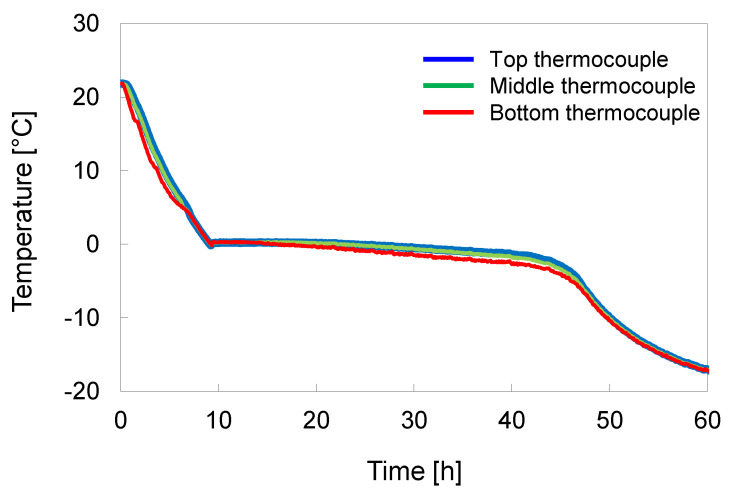
Evolution of specimen temperature during freezing. Thermocouples are installed on the top, middle, and bottom of the specimen to monitor the direction of cooling/freezing.

The specimen temperature evolution measured at three separate locations (top, middle, and bottom) is summarized in Figure 2.

The bottom temperature is lower than that of the middle and top, which indicates that the freezing direction is sufficiently induced upwards. No significant changes in the height of the specimen were observed during freezing. After freezing the specimens, the frozen specimens were removed from the freezing mold and trimmed to 100 mm in height and 50 mm in diameter using an electric grinder for the following triaxial compression test. Note, the aforementioned trimming process was performed in the walk-in freezing chamber to prevent the unintended or uncontrolled thawing of the specimens.

### 2.2. Test Procedures

The prepared frozen specimens were placed on the pedestals for the triaxial test. A vacuum pressure of 20 kPa was applied to the specimen using an evacuation chamber to minimize the air space between the specimens and membrane. While maintaining the vacuum pressure, de-aired cold water (close to 0 °C) was injected into the cell. The specimen vacuum pressure was released after the cell was filled with de-aired cold water to control the cell pressure. A minimum cell pressure of 20 kPa was selected to ensure that the specimen could stand-alone after completing the thawing phase. This process was performed within six min to avoid uncontrolled thawing [2].

Table 1 summarizes the test conditions depending on the drainage condition during thawing and on the test phase (i.e., consolidation phase) to bring the specimen to the in situ stress state (100 kPa in this study).

The prepared specimens were thawed under undrained (UT) and drained (DT) conditions with various degrees of cell pressure (i.e., 20, 50 and 100 kPa) to evaluate the effects of drainage and confining pressure on the specimen’s disturbance. After completing the thawing phase under the target pressure, the saturation phase was conducted to fully saturate the specimens (B-value ≥ 0.95 in this study). Because the initial vertical stress was 100 kPa during freezing, the target stress during the consolidation phase was set to 100 kPa, except for one case (DT20-C150), to simulate the initial stress condition. For the DT50-C100 specimen, as an example, a pressure of 50 kPa was applied during the thawing phase, and the remaining 50 kPa was applied to the specimen during consolidation to have the specimen finally subjected to a confining pressure of 100 kPa before the shear phase. The shear phase ensues after no significant volume changes occurred in the specimen under the target confining pressure. During the shear phase, the compressional axial strain was applied to the specimen at a strain rate of 0.2%/min.

### 2.3. Measurement System

**Figure 3:**
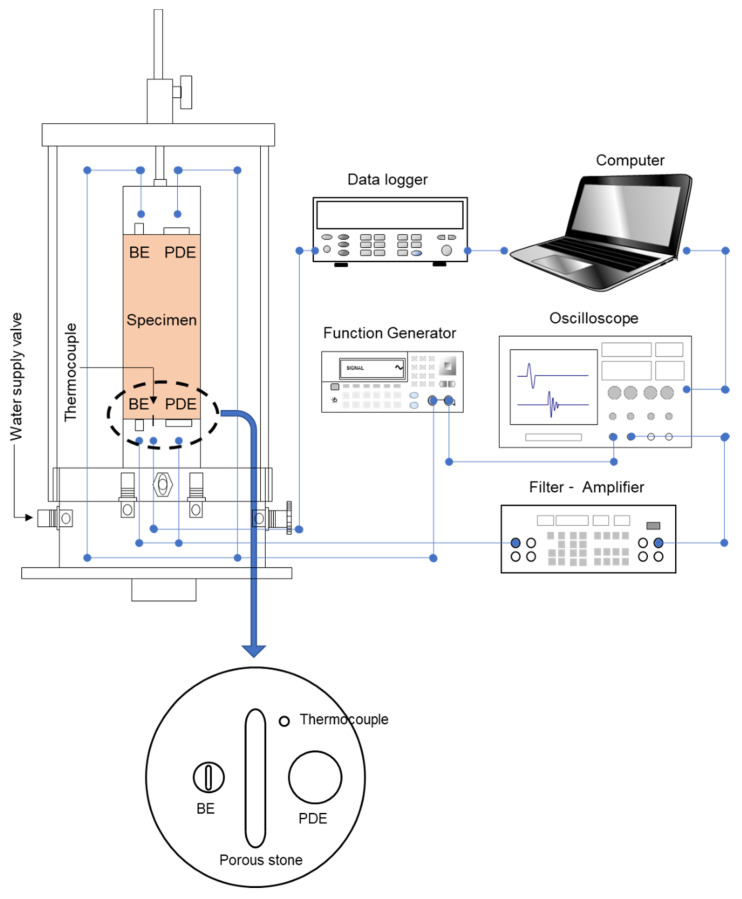
Measurement system during the triaxial test. The wave sensors (i.e., bender element (BEs) and piezo disk element (PDEs)) and thermocouple are equipped on the top and bottom pedestals.

Figure 3 presents a schematic drawing of the triaxial test system for monitoring the P- and S-wave signals and temperature.

The piezo disk elements (PDEs) and bender elements (BEs) are equipped on the top and bottom pedestals to measure the P- and S-wave velocities. The PDEs are 12 mm in diameter and 0.4 mm thick, and the BEs are 6 mm long, 4 mm wide, and 0.6 mm thick. The bender elements are extruded as a cantilever beam to have better contact with the specimen. Note, BEs have been widely used to measure the small-strain shear stiffness owing to the effective coupling between the soils and sensors.

The input signal is generated from a function generator (Keysight 33220A, Keysight Technologies, Santa Rosa, CA, USA) and transported to source sensors. The wave signal propagated through the specimen is captured on the receive sensors and then transmitted to a filter-amplifier (Krohn-Hite 3361, Krohn-Hite Corporation, Brockton, MA, USA). The processed signal is presented and saved in an oscilloscope (Keysight DSOX2014A, Keysight Technologies, Santa Rosa, CA, USA). The elastic wave velocity is the ratio of the travel length to travel time, and the travel lengths are the plate-to-plate and tip-to-tip distances for the PDEs and BEs, respectively. A thermocouple wire (K-type) was installed on the bottom pedestal to monitor the temperature. Porous stones were mounted on the center of the top and bottom pedestals to enable uniform pore-water drainage during the test.

## 3. Results

### 3.1. Changes in Temperature during Thawing

**Figure 4:**
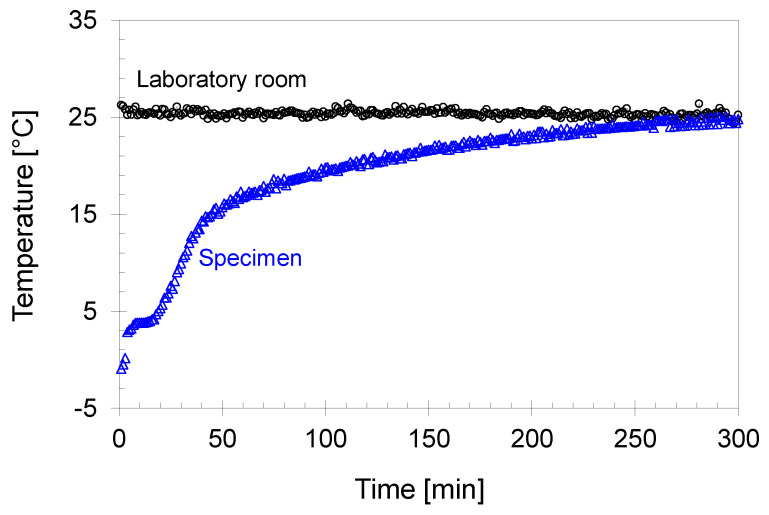
The measured temperature of the tested specimen (DT20-C100) during thawing. The specimen was placed on the pedestal of the triaxial system and was subjected to a 20 kPa confining stress. The cell was filled with cold water to avoid the sudden/uncontrolled melting of the specimen, and then were in equilibrium at room temperature.

The specimen temperature was continuously measured during thawing, as shown in Figure 4.

Note, the temperature was measured at the bottom of the specimen. After the cell was filled with cold de-aired water, the specimen temperature continuously increased to room temperature at 25 °C for the equilibrium of the cell water temperature at room temperature. Because the heat exchange between the cell water and soil specimen was the main contributor to thawing the specimen, the thawing phase continued until the specimen temperature reached room temperature. The UT specimen remained in the undrained condition during thawing, whereas the DT specimen circulated the pore water while maintaining the target pore pressure.

### 3.2. Undrained Triaxial Test

**Figure 5:**
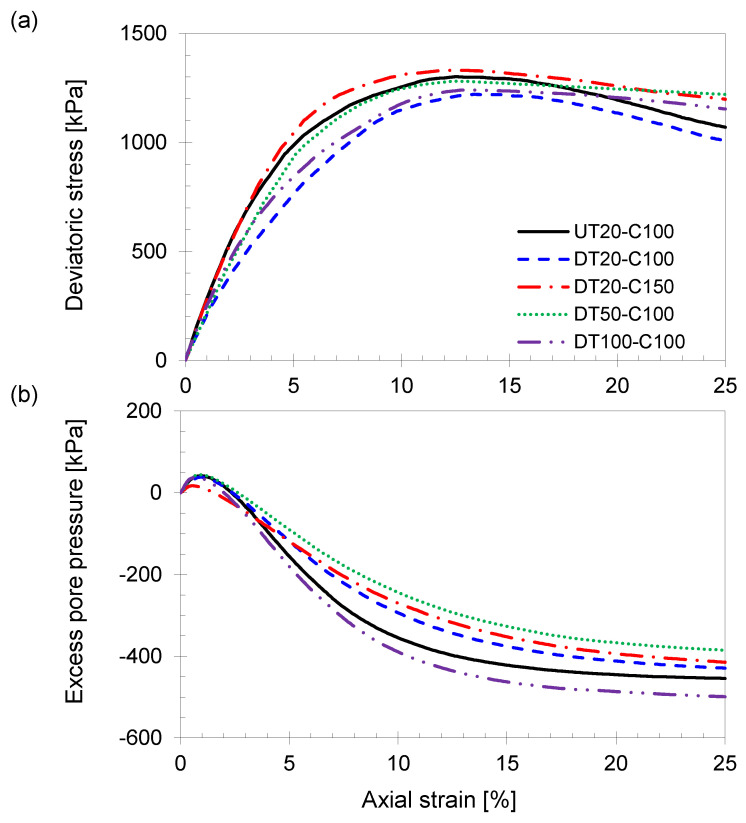
Results of undrained triaxial compression tests. The shear phase continues until the axial strain reached 25%. (**a**) Deviatoric stress. (**b**) Excess pore pressure.

The results of the undrained triaxial compression tests are presented in Figure 5. All the tested specimens revealed a similar trend of the deviatoric stress and generation of negative excess pore-water pressure following a slight increase in excess pore-water pressure during the early shear phase (axial strain < 1%). Table 2 summarizes the calculated engineering properties and parameters as a result of the undrained triaxial compression test.

The UT20-C100 specimen has a 13% higher shear strength than that of the DT20-C100 specimen, indicating that the UT condition during thawing causes a higher shear strength. Comparing the shear strengths of specimens DT20-C100, DT50-C100, and DT100-C100 demonstrates that the greater stress restoration portion of the initial vertical stress during the thawing phase can overestimate the shear strength of the thawed specimen. The DT20-C150 specimen, which was thawed under the DT condition and restored more extensive stress than the initial stress condition, demonstrated the most overestimated shear strength.

### 3.3. Elastic Wave Measurement

#### 3.3.1. Saturation Phase

**Figure 6:**
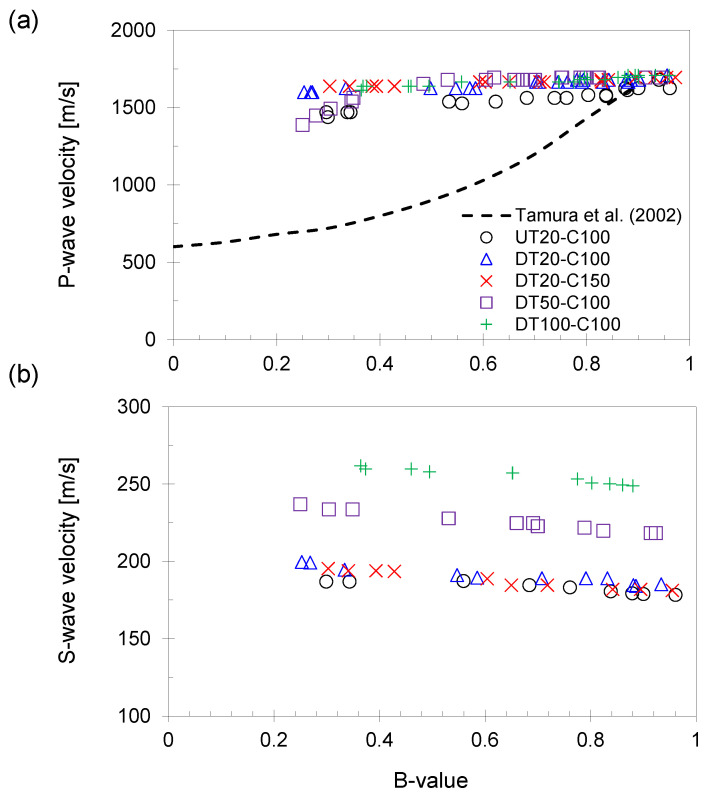
The measured P- and S-wave velocities during the saturation phase. The specimens were subjected to different degrees of confining stress. (**a**) P-wave velocity versus B-value. The two lines indicate the relationship between the B-value and P-wave velocity [12]. (**b**) S-wave velocity versus B-value.

The P- and S-wave signals were continuously captured during the saturation phase. The relationships of the calculated P- and S-wave velocities with the B-value are shown in Figure 6.

After the thawing phase, the P-wave velocity of the specimens ranged from 1300 to 1600 m/s. The P-wave velocity increased up to 1700 m/s after completing the saturation phase (i.e., the B-value is greater than 0.95). Note, the P-wave velocity of water is approximately 1500 m/s [13].

Previous studies have shown that the P-wave velocity is lower than 1000 m/s when the B-value is lower than 0.6 [12]. However, in this study, a higher P-wave velocity was determined despite the B-values being below 0.6. The compressional wave velocity may be affected by the air bubble size and location [12]. Because the P-wave velocity is significantly affected by the compressibility of the soil skeleton and pore materials, the P-wave can pass through a relatively higher rigid path when the air bubbles are unevenly distributed inside the specimens. Thus, the P-wave velocity could be as high as 1500 m/s at a low B-value [14].

The S-wave velocity responses during the saturation phase are summarized in Figure 6b. At the beginning of the saturation phase, the S-wave velocity varies because the specimens were subjected to various degrees of confining stress and drainage conditions during the previous thawing phase. Larger confining stress during the thawing phase results in a higher S-wave velocity. As the B-value increases, the S-wave velocities of all specimens exhibit either relatively constant or slightly decreasing values. Tamura et al. proposed that the B-value has no significant effect on the S-wave velocity [12]. Based on the definition of the B-value, which is the ratio of the excess pore-water pressure to the applied external pressure, a small amount of air content can cause a considerable reduction in the B-value owing to the high bulk compressibility of air or gas. Nevertheless, it may not significantly affect water saturation owing to the fact that higher saturation does not guarantee higher B-values.

In this study, the tested specimens had lower B-values at the beginning of the saturation phase; however, the specimens consisted of a high degree of water saturation (i.e., close to 100% from the definition). Thus, the S-wave velocity changes during the saturation phase in this study are related to the B-value rather than water saturation. However, the reasons for the slight decrease in the S-wave velocity are unclear. Presumably, the repeated fluctuations of the confining pressure to measure the B-value (i.e., 20 kPa increments of cell pressure, then back to the initial value) under the UT conditions may cause a weakened soil skeleton.

#### 3.3.2. Consolidation Phase

**Figure 7:**
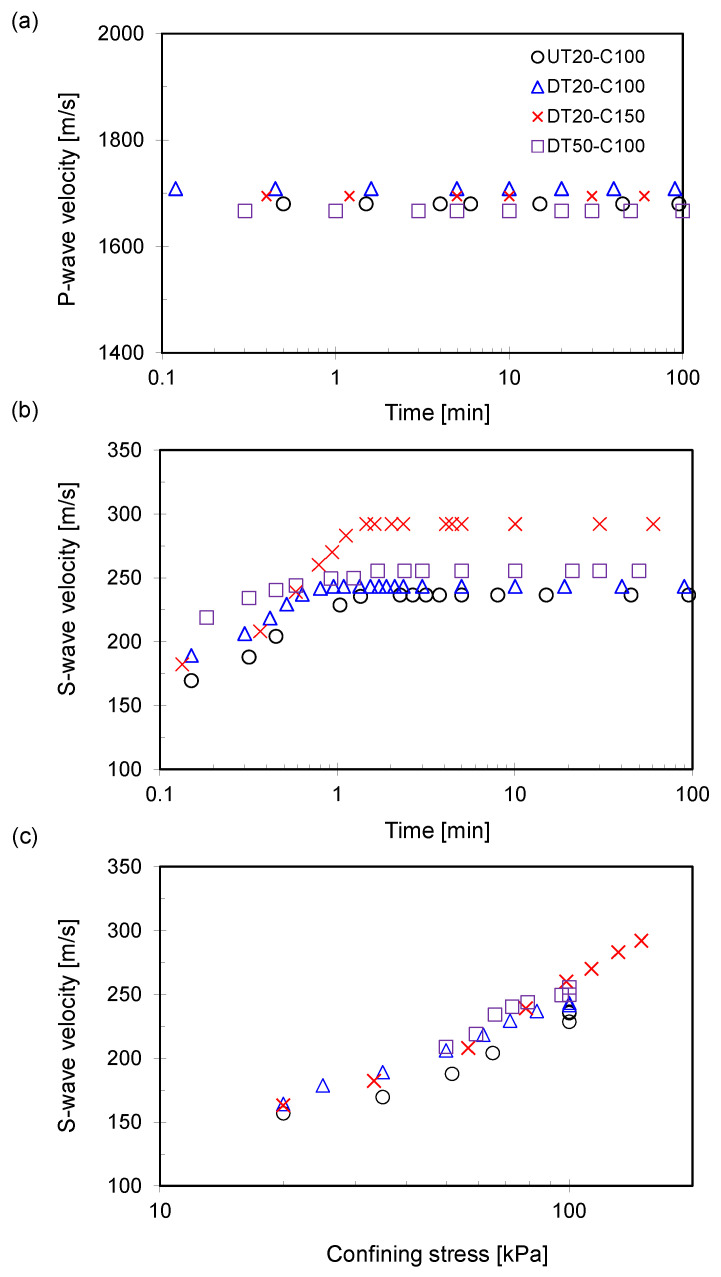
Measured P- and S-wave velocities during the consolidation phase. The stress rate is 100 kPa/min. (**a**) P-wave velocity versus time. (**b**) S-wave velocity versus time. (**c**) S-wave velocity versus confining stress. Note, the DT20-C150 specimen is subjected to 150 kPa as the confining stress.

Figure 7 presents the evolution of the P- and S-wave velocities during the consolidation phase.

In the consolidation phase, two confining stress levels (i.e., 150 kPa for DT20-C150 and 100 kPa for the others) were applied to the specimens at a rate of 100 kPa/min. The consolidation phase was skipped for the DT100-C100 specimen because the target confining stress was accomplished during the thawing phase. The P-wave velocity evolution is presented in Figure 7a, and no significant changes were observed, although the confining effective stress increased. This result indicates that the P-wave velocity of the fully saturated specimens is primarily dominated by the pore-water compressibility rather than the confining pressure (≤150 kPa in this study).

The calculated S-wave velocity was plotted according to the elapsed time (Figure 7b) and the confining stress (Figure 7c). Because the stress rate was 100 kPa/min, most of the target confining stress was achieved within 1 to 1.5 min. The consolidation phase is critical because the initial stress conditions prior to freezing can be restored to the specimens after the freezing and thawing processes. Although the UT20-C100 and DT20-C100 specimens have different drainage conditions during the thawing phase, no significant differences in the S-wave velocity are observed, indicating that the drainage condition effects on the small-strain shear stiffness are limited. The DT20-C150 specimen has the highest S-wave velocity after completing the consolidation phase because it is primarily affected by the effective stress condition. The S-wave velocity of the DT50-C100 specimen exhibited higher values than the UT20-C100 and DT20-C100 specimens, demonstrating that the higher confining stress during the thawing phase can make the specimen more rigid, despite undergoing identical confining stress conditions. The DT100-C100 specimen has an S-wave velocity of 250 m/s after saturation (i.e., under 100 kPa of confining stress), as shown in Figure 6b, which is higher than that of the S-wave velocity of the UT20-C100 and DT20-C100 specimens.

#### 3.3.3. Shear Phase

**Figure 8:**
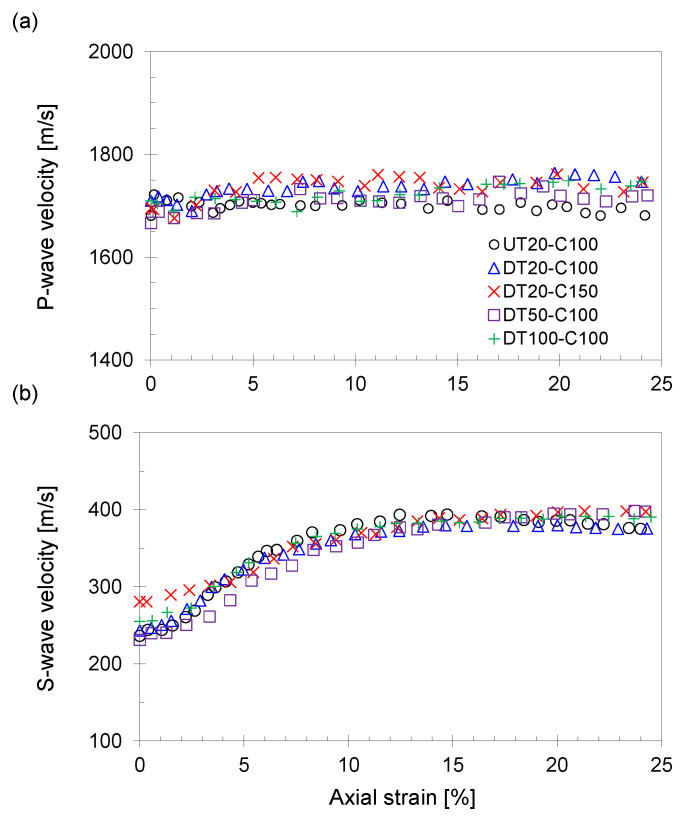
Measured P- and S-wave velocities during the shear phase. (**a**) P-wave velocity versus axial strain. (**b**) S-wave velocity versus axial strain.

The P- and S-wave velocities during the shear phase are presented in Figure 8.

Figure 8a indicates that the P-wave velocities of all specimens are nearly constant, regardless of the axial strain, which indicates that thawing and stress restoration methods have no significant effect on the P-wave velocity. This result also confirms that the P-wave velocity is primarily affected by the pore-filling constituents. Figure 8b indicates the evolution of the S-wave velocity during the shear phase. As the shear strain increases, both vertical and horizontal effective stresses increase until the shear strain reaches 12–15%. Because the S-wave velocity is primarily affected by the effective stress status, the measured S-wave velocity increases until shear failure and then remains constant for the following shear phase (Figure 5).

### 3.4. Cyclic Simple Shear Test

Soil liquefaction occurs when the soil loses its stiffness and strength due to increased pore-water pressure and corresponding reduced effective stress [15]. Soil liquefaction is more pronounced in loosely packed deposits owing to the contractive soil behavior during cyclic shear loading. The direct simple shear test has been widely used in geotechnical engineering to mimic field-loading conditions, especially when the principal stresses are rotated during the shearing process [16,17,18].

In this study, complementary monotonic and cyclic simple shear tests were performed to estimate the packing density effects on the cyclic response in sandy soils. The specimens were sheared under constant volume conditions during monotonic and cyclic loading. In the constant volume condition, the specimen shearing response can be considered as the fully undrained condition [19], and the changes in the effective vertical stress correspond to the pore-water pressure development [20,21,22].

Specimens were prepared using Ottawa sand, which has an identical grain size distribution to Jumunjin sand 30/50 used for the triaxial compression test. The oven-dried sand was mixed at a 2% moisture content to aid compaction and was placed into a mold in three layers. A shearing ring stack was installed on the specimens to prevent lateral direction displacement. After the three layers were compacted, the top cap was placed on the specimen, and the height of the specimen was determined using electronic calipers.

The specimens were packed into two different relative densities (i.e., approximately 50% for loose packing and 80% for dense packing). Deionized water circulated the specimen upwards until no air bubbles were observed on the outlet. The specimens were then subjected to 100 kPa of vertical stress without lateral deformation (i.e., *K*_0_ consolidation). Monotonic shear tests were conducted at a shearing rate of 0.0635 mm/min. The cyclic shear tests employed an identical shear rate for the monotonic test, and the appropriate cyclic stress ratio (CSR) was determined using the monotonic peak shear stress level. The liquefaction criteria are met here when a 7.5% double amplitude shear strain is achieved [23,24]. The details of the test conditions are listed in Table 3.

**Figure 9:**
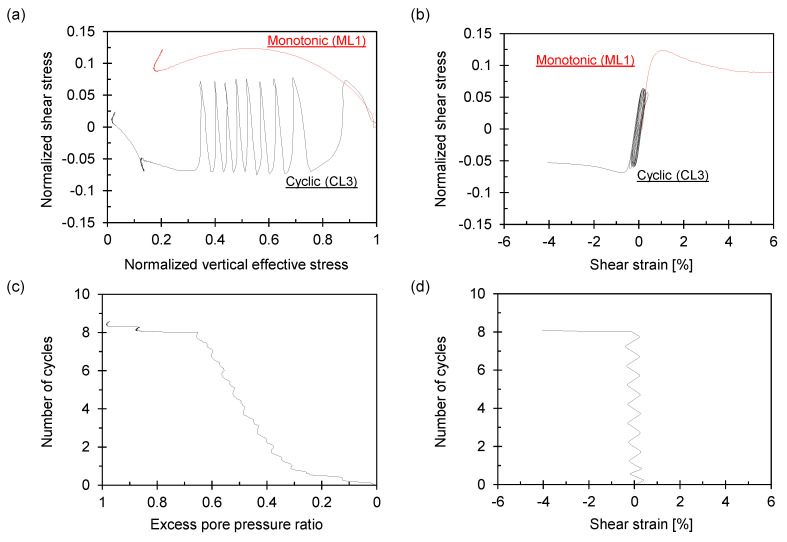
Cyclic simple shear results for loose sample *D_r_* ≈ 50%. Stresses are normalized by initial vertical stress (*σ_v_* = 100 kPa). (**a**) Normalized shear stress versus normalized vertical effective stress. (**b**) Normalized shear stress versus shear strain. (**c**) The number of cycles versus excess pore pressure ratio. (**d**) The number of cycles versus shear strain.

**Figure 10:**
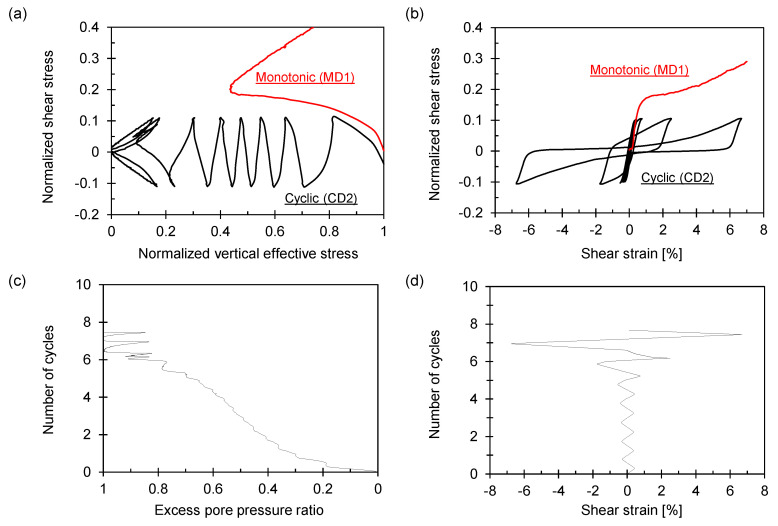
Cyclic simple shear results for dense sample *D_r_* ≈ 83%. Stresses are normalized by initial vertical stress (*σ_v_* = 100 kPa). (**a**) Normalized shear stress versus normalized vertical effective stress. (**b**) Normalized shear stress versus shear strain. (**c**) The number of cycles versus excess pore pressure ratio. (**d**) The number of cycles versus shear strain.

The monotonic and cyclic simple shear test results are presented in Figure 9 and Figure 10.

With the increase in the number of cycles shown in Figure 9a and Figure 10a, the vertical effective stress continuously decreases and finally loses nearly all the stress. The shear stress response of the first cycle until the targeted shear stress follows the monotonic shear response because the test conditions (i.e., packing density and shear rate) are identical. Compared to the dense specimen shown in Figure 10, the loose specimen in Figure 9 exhibits a rapid shear deformation in the last cycle, indicating a sudden drop in the vertical effective stress. Loose granular specimens may have significantly softened during cyclic loading, resulting in a large flow deformation [25], as depicted in Figure 9b,d. The pore-water pressure gradually developed as the cyclic loading continued, reaching the initial vertical effective stress when liquefaction occurred, as shown in Figure 9c.

## 4. Discussion

### 4.1. Effects of Drainage and Stress Recovery Conditions

#### 4.1.1. Elastic Wave Velocity

The measured wave velocities at each test phase are summarized in Table 4. The P-wave measurement is not available prior to freezing; however, the specimens are fully saturated because the velocity after thawing is close to 1500 m/s. Prior to freezing, the specimen S-wave velocity was approximately 240 ± 5 m/s under the vertical stress of 100 kPa. During the consolidation phase, which simulates the in-situ conditions, the results demonstrate that the S-wave velocities of the UT20-C100 and DT20-C100 specimens were closest to those of the initial unfrozen specimens. The results of the DT50-C100 and DT100-C100 specimens suggest that stress restoration during the thawing phase can make the specimens more rigid, resulting in a higher S-wave velocity and shear strength.

#### 4.1.2. Axial Deformation

The measured axial deformation during the thawing, saturation, and consolidation phases are summarized in Table 5. The initial height of the frozen specimens is approximately 100 mm. Axial deformation was used rather than volume change because the frozen specimen should be thawed for laboratory testing. It is challenging to accurately measure the volume changes generated only from specimens [2]. In addition, an accurate volume change cannot be obtained when filling the cold water in the space between the frozen specimens and the membrane. Thus, axial deformation is an alternative indicator of the sample disturbance of the thawed specimen.

The results reveal that the UT20-C100 specimen has considerably more axial deformation than the others, indicating that UT thawing causes more substantial volume changes. Although the DT20-C150 specimen is subjected to higher confining stress during the consolidation phase, the axial deformation is less than that for the UT20-C100 specimen. This result suggests that axial deformation primarily occurs during the thawing phase, and the axial strain generated during the consolidation phase is insignificant for sand. The DT50-C100 and DT100-C100 specimens, which were subjected to 50% and 100% of the initial stress during thawing, suggest that applying a substantial portion of the in situ stress during the thawing phase can generate greater axial deformation. Furthermore, significant lateral deformation (shrinkage) may ensue when higher confining stress is applied during the thawing phase because the specimens are multi-directionally thawed. A non-uniform stress distribution produced by the uneven thawing of the specimen surface under higher confining stress can generate an unrestrained disturbance [2].

Based on the elastic wave and axial deformation measurement results, the DT20-C100 specimen, which is thawed in the DT condition and restored to the initial stress conditions during the consolidation phase, exhibited less sample disturbance compared to the initial unfrozen specimens. Multidirectional thawing under compressive conditions may cause higher volume changes owing to the concentration and uneven stress distribution [8]. The results also suggest that a minimum confining stress is necessary to help the specimen stand-alone during thawing, and the remaining stress should be restored during the consolidation phase. In addition, the shear strength of the DT20-C100 specimen was lower than that of the other specimens (Figure 5a), suggesting that an inappropriate test method for the frozen specimen overestimates the shear strength.

### 4.2. Sand Liquefaction Response

**Figure 11:**
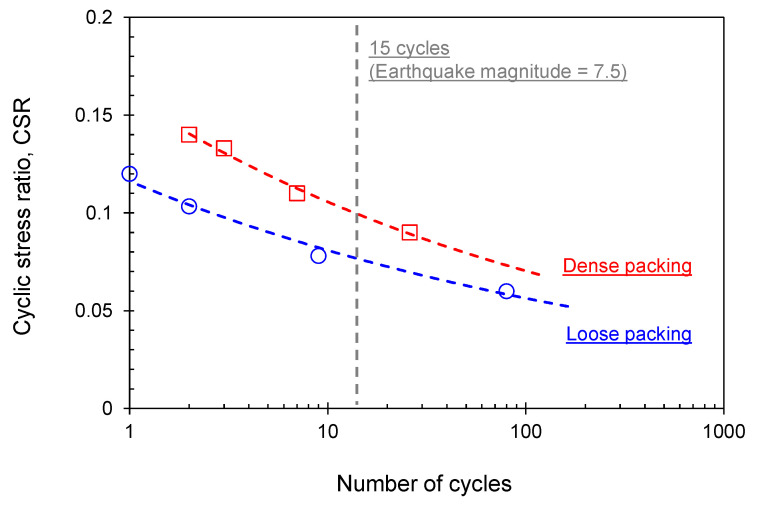
Cyclic stress ratio versus the number of cycles to liquefaction. The inserted reference bar indicates the 15 cycles corresponding to the earthquake magnitude 7.5.

Figure 11 summarizes the relationship between the cyclic stress ratio (CSR) and the number of cycles required for a 7.5% double amplitude shear strain for loose and dense specimens.

The dense specimens require a higher number of cycles within a given CSR range to trigger liquefaction. It indicates that the dense specimens are less susceptible to liquefaction than loose packing specimens because densification is beneficial for liquefaction resistance. During the triaxial test process of the undisturbed frozen specimens, the specimens may have experienced volume contraction (i.e., axial deformation) caused during the thawing, saturation, and consolidation phases (Section 4.1). In this study, the developed axial deformation causes an increase in the relative density by up to 10%, depending on the thawing and initial stress restoration process. Thus, volume changes in frozen-thawed specimens (i.e., disturbance in artificial frozen-thawed sandy soils) should be considered for all test protocols; otherwise, the unrestrained volume contraction of sandy materials causes an overestimation of the liquefaction resistance.

## 5. Conclusions

This study aims to estimate the effects of the thawing and in situ stress restoration process on the stiffness, strength, and liquefaction resistance of artificial frozen sandy soils. Specimens were prepared in a freezing mold with a relative density of 60% and frozen at −20 °C for 60 h under the vertical stress of 100 kPa. The specimen undergoes thawing, saturation, and consolidation phases with various drainage and confining stress conditions, and the shear phase is sequentially performed. The temperature is monitored during the thawing phase, and axial deformation and elastic wave signals are measured during the entire protocol. In addition, monotonic and cyclic simple shear tests using sandy soils with a relative density of 50% and 80% were also conducted to determine the packing density effect on liquefaction resistance. The main observations are as follows:

- The P-wave velocity increases up to 1700 m/s during the saturation phase. It is maintained constant during the consolidation and shear phases because fully saturated sand is primarily dominated by pore-filling constituents (i.e., pore water).
- The S-wave velocity varies throughout the phases with respect to the change in effective stress, which predominantly affects the small-strain shear stiffness of the sand. By comparing the S-wave velocities before freezing and after consolidation, the specimen subjected to 20 kPa during thawing and 100 kPa during consolidation (UT20-C100 and DT20-C100) showed the most suitable protocol for initial stress restoration.
- Axial deformation, selected for assessing volume change, is significantly generated by undrained or applied by greater confining stress conditions during thawing. Likewise, the shear strength of DT20-C100 was lower than that of the other drainage and confining stress conditions.
- Based on the axial deformation and shear strength, thawing in drained conditions and restoring most of the initial stress during the consolidation phase can minimize the disturbance of sands. Consequently, a lower disturbance in frozen-thawed sand prevents the overestimation of liquefaction resistance, which can be enhanced in densely packed conditions.

## Figures and Tables

**Table 1:** Triaxial compression test condition.

Specimens	Thawing Phase		Consolidation Phase	Restoration of the Initial Vertical Effective Stress (100 kPa)
	Drainage	Confining Stress (kPa)	Confining Stress (kPa)	
UT20-C100	Undrained	20	100	at the consolidation phase
DT20-C100	Drained	20	100	at the consolidation phase
DT20-C150	Drained	20	150	at the consolidation phase (*σ_c_* = 150 kPa)
DT50-C100	Drained	50	100	at the thawing and consolidation phase (50 kPa/100 kPa)
DT100-C100	Drained	100	100	at the thawing phase

UT = undrained thawing, DT = drained thawing, C = consolidation, and each number indicates the degree of confining stress in kPa at each test phase (i.e., thawing or consolidation). Initial vertical effective stress during freezing: 100 kPa (for all tested specimens). Sample size: 50 mm diameter and 100 mm height. The relative density of specimens is approximately 60%.

**Table 2:** Result of the undrained triaxial compression test.

Specimens	Axial Strain at Failure (%)	Deviator Stress at Failure (kPa)	Shear Strength at Failure (kPa)	Mean Effective Stress at Failure (kPa)	Excess Pore Pressure at Failure (kPa)	Friction Angle at Failure (^o^)
UT20-C100	12.5	1308.2	703.1	931.8	−397.9	33.0
DT20-C100	13.3	1225.3	621.8	862.6	−355.7	32.2
DT20-C150	12.1	1336.5	712.4	804.6	−311.2	33.3
DT50-C100	12.9	1240.5	645.2	954.6	−441.0	31.9
DT100-C100	12.5	1281.7	693.3	819.6	−292.7	32.8

**Table 3:** Details of the cyclic simple shear test.

Test ID	Relative Density (%)	Specimen Height (mm)	Cyclic Stress Ratio, CSR	Number of Cycles	Note
ML1	51.8	18.2	-	-	Monotonic shearing
CL1	52.1	18.2	0.103	2	
CL2	49.4	18.3	0.050	-	No liquefaction until 200 cycles
CL3	49.9	18.3	0.070	9	
CL4	52	18.2	0.120	1	
CL5	53.5	18.2	0.060	80	
MD1	83.2	17	-	-	Monotonic shearing
CD1	83.6	17	0.130	3	
CD2	83.7	17	0.110	7	
CD3	82.4	17.1	0.090	26	
CD4	81.9	17.1	0.140	2	

ML = monotonic loading for loose packing, CL = cyclic loading for loose packing, MD = monotonic loading for dense packing, and CD = cyclic loading for dense packing. The number of cycles = required number of cycles to trigger 7.5% double amplitude shear strain.

**Table 4:** Measured P- and S-wave velocities during the test.

Specimens	P-Wave Velocity (m/s)				S-Wave Velocity (m/s)			
	Before Freezing	After Thawing	After Saturation	After Consolidation	Before Freezing	After Thawing	After Saturation	After Consolidation
UT20-C100	N/A	1369.9	1680.7	1680.7	240 ± 5	176.4	178.3	236.4
DT20-C100		1600.0	1709.4	1709.4		183.5	185.2	243.3
DT20-C150		1639.3	1694.9	1694.9		180.3	181.2	280.9
DT50-C100		1388.9	1666.7	1666.7		226.2	218.3	255.4
DT100-C100		1612.9	1709.4	1709.4		251.9	248.8	248.8

**Table 5:** Measured axial deformation during the thawing, saturation, and consolidation phases.

	Undrained Thawing	Drained Thawing			
	UT20-C100	DT20-C100	DT20-C150	DT50-C100	DT100-C100
Axial deformation (mm)	1.98	1.15	1.24	1.21	1.62

## Data Availability

The data presented in this study are available on request from the corresponding author. The data are not publicly available due to ongoing research project.
